# Cross-Sectional Study on Health Literacy and Internet Accessibility Among Patients With DM in Gansu, China

**DOI:** 10.3389/fpubh.2021.692089

**Published:** 2021-10-13

**Authors:** Na Zhao, Xifeng Luo, Hailiang Zhang, Runjing Dai, Weimin Pan, Brett D. Hambly, Shisan Bao, Xiangdong Zhu, Jingchun Fan

**Affiliations:** ^1^Centre for Evidence-Based Medicine, School of Public Health, Gansu University of Chinese Medicine, Lanzhou, China; ^2^Hospital Infection-Control Department, Affiliated Hospital of Gansu University of Chinese Medicine, Lanzhou, China; ^3^Department of Public Health, Pingliang Second People's Hospital, Pingliang, China; ^4^Department of Mental Health, Gansu Provincial Center for Disease Control and Prevention, Lanzhou, China; ^5^Center for Health Futures, Torrens University Australia, Sydney, NSW, Australia; ^6^School of Traditional Chinese Medicine, Ningxia Medical University, Yinchuan, China; ^7^Basic Medical School, Gansu University of Chinese Medicine, Lanzhou, China

**Keywords:** China, DM, health literacy, knowledge, attitude, practice, internet accessibility

## Abstract

**Objective:** To determine the relationship between the health literacy of patients with diabetes mellitus (DM) and the accessibility of internet surfing for information concerning DM.

**Methods:** A multistage stratified sampling method was utilized to conduct a questionnaire survey on DM health literacy and internet accessibility among 1,563 patients with DM in Gansu Province in 2020. Logistic regression was performed to analyze the factors that influence health literacy and internet accessibility; while the chi-square test was used to compare the differences in needs.

**Results:** Among 1,563 valid questionnaires collected with an effective rate of 95.7%, there were 65.4, 66.3, or 51.1% of patients with DM were found to have good health knowledge, attitudes, or practice levels, respectively. Occupation, income, disease course of DM, and accessibility to the internet were the main factors influencing health literacy. Age, residency, occupation, education, income, and family history of DM were the factors influencing accessibility to internet surfing for DM. The expectations from patients with DM for the capacity to obtain DM information from traditional sources or through internet sources was 1,465 (93.7%) or 1,145 (73.3%), respectively. Patients with DM had a 2-fold higher desire to obtain DM health information from internet media if the patients had access to the internet than those without (*P* < 0.05).

**Conclusions:** The socioeconomic status and access to the internet were the main contributing factors for health literacy, as socioeconomic status is closely related to access to the internet.

## Introduction

Diabetes mellitus (DM) is one of the major fast growing non-communicable disease (NCD) threats to global public health (1). The International Diabetes Federation (IDF) reports that there were 463 million adults with DM in 2019 and estimates it will reach 700 million in 2045 (2). Similarly, the number of patients with DM has increased from 110 million in 2017 (3) to 116 million in 2019 in China (4), and the estimated number will be 120 million by 2045 (5). Gansu Province, located in the Northwest of China, is relatively underdeveloped with the lowest GDP in China, mainly due to its geographic location (6). Consequently, the level of education and the opportunities for employment are relatively low (7), but the situation has been rapidly improving over the last few decades. It is highly concerning that the prevalence of DM in Gansu has increased from 3.2% in 1999 (8) to 10.6% in 2019 (9), which has attracted extremely high attention from the local health authority.

It has been well-documented that knowledge, positive attitudes, and self-management skills of patients, together with lifestyle choices, are essential for proper control/maintenance of blood glucose levels (10). The WHO has defined health literacy as personal characteristics and social resources needed for individuals and communities to access, understand, appraise, and use information and services to make decisions about health (11). DM health literacy is considered to be an essential part of DM self-management (12). There are two approaches to improve health literacy, i.e., the use of traditional paper and/or public broadcasting, and the use of more recently available internet resources. Internet surfing is a relatively common and easy source to obtain health information about the development and management of DM (13). This is in line with the findings from the general population, which show that the majority of internet users surf the internet to understand the medical conditions that relate to their own needs, e.g., clinical symptoms and signs, and many will also try to self-diagnose (14).

Following the substantial development of the internet over the last few decades, particularly among developed countries, the pattern by which patients with DM obtain DM-related information has changed, i.e., more patients with DM are seeking health literacy from internet resources due to its convenience and availability. For example, 90% of internet users in the United States of America sought health information online in 2016 (15); whereas the proportion of internet-active users worldwide has reached 59.5% as of January 2021 (16). The accessibility and use of internet surfing have also improved over the last two decades in China. The internet penetration rate in China has gradually increased from 46% in 2013 (17) to 52% in 2016 (18), but at present, it is only 39% in Gansu Province. Internet surfing also enables patients with DM to interact with each other and with other social networks, particularly to share self-management and awareness of DM (19). This is supported by the finding that there is a correlation between internet surfing for DM health information and management of blood glucose in patients with DM, especially impacting the improved quality of life (20). In addition, patients with DM improve their health status *via* internet surfing as a consequence of their improved literacy concerning self-management of DM (21), as well as the capacity to obtain different opinions from different web-based media options (22).

However, the expectations of patients with DM and the availability of resources from public platforms in areas of relatively low economic income within the regions of China remain to be explored. Thus, the aim of the current study was to determine the relationship between the status of health literacy of patients with DM and the accessibility of internet surfing for DM within areas of lower socioeconomic status within China.

## Materials and Methods

### Methods

The study was designed as a questionnaire-based cross-sectional analysis. A questionnaire survey was carried out among 1,563 patients with DM in Gansu Province in July 2020, using a multistage stratified sampling method. The selection criteria for the respondents were as follows: (1) fit the diagnostic criteria for DM (23), (2) age was ≥18 years, (3) cognitive level was adequate to respond to the questions, (4) voluntary. All of the respondents gave informed consent. The exclusion criteria were if the survey could not be completed due to a physical and/or psychological disorder, or if the respondent was pregnant or breastfeeding. Each questionnaire was completed *via* a face-to-face, in-home survey, with telephone follow-up by trained investigators. Any missing or unclear items within the questionnaire were supplemented with a follow-up telephone call to minimize deviation and impact on the study results.

### Source of Patients

All the patients who received a diagnosis of DM are recorded electronically in the Public Health Care System from the Health Service Centers in the clinics from communities and/or villages in Gansu Province. Although the electronic database records the entire patient cohort attending clinics amongst the regular residents of their respective regions, including their diagnosis of DM, a few patients with DM may have been missed out from the current study because some of them with DM may have moved out of the area to undertake relatively temporary job opportunities outside the regions or even out of the province. In addition, it is also possible that a few people might be at the preclinical phase or be undiagnosed patients with DM, who could be missing from the data source.

### Sampling and Sample Size

**Figure 1:**
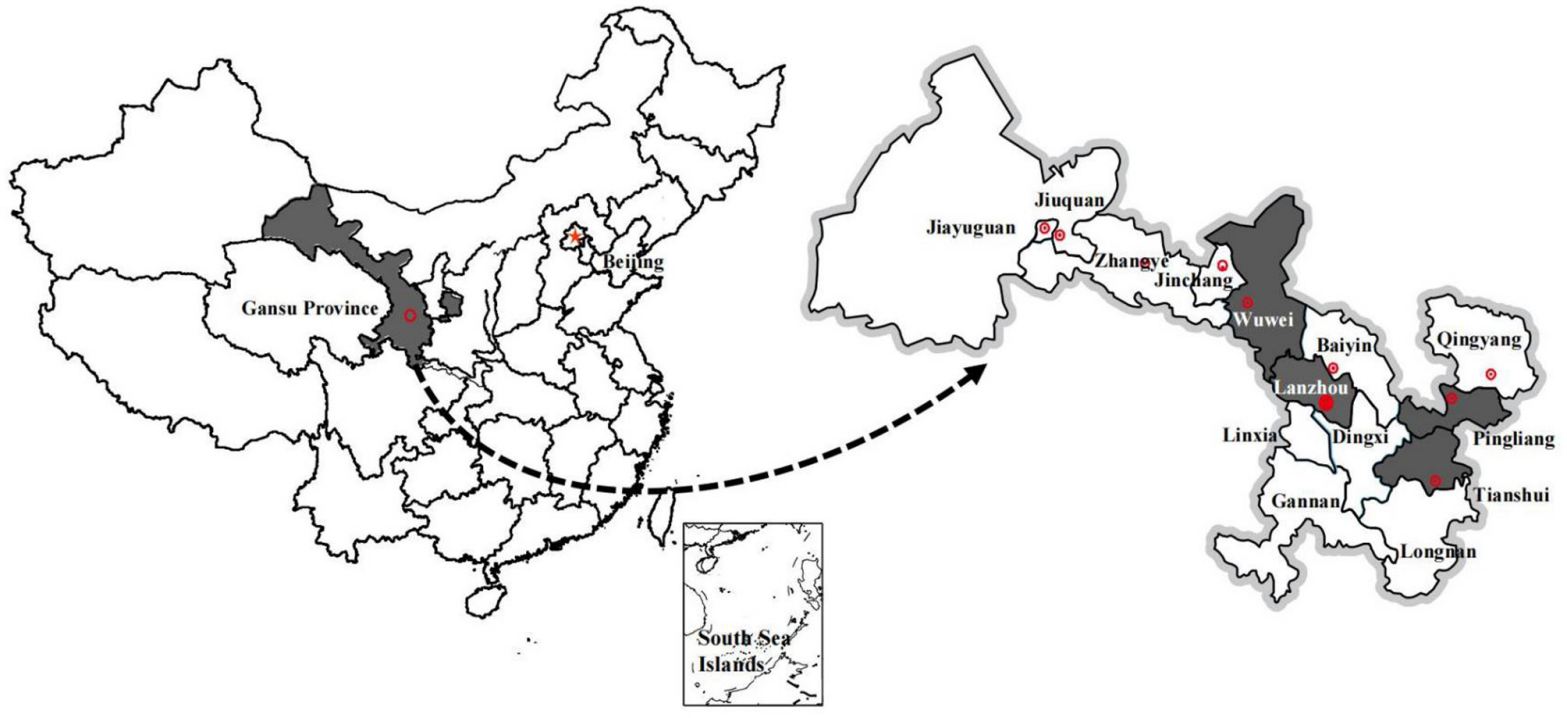
The regional distribution of sample cities in Gansu Province.

In the current study, multistage stratified sampling was performed according to the administrative division level in China, i.e., sequentially as expressed: prefecture (city), county (district), township (subdistrict), and village (community) in Gansu Province (24). Based on the Board of Medical and Health Service System Planning (2016–2020), Gansu Province is divided into four medical zones with similar populations (Lanzhou, Pingliang, Tianshui, and Wuwei) (25) at level one (prefecture) (Figure 1). Within these four regions, one district or one county was selected randomly as a representative for either the urban or the rural population, constituting the second level (county). Then one subdistrict or one township was randomly selected as the third level (township). Finally, five community health service centers were also randomly selected (the most basic level of medical service) as level 4 (village). Thus, a total of 20 community health service centers from towns and 20 village clinics from rural villages were selected. The prevalence of DM in urban or rural residents in Gansu Province was 11.5 or 9.5%, respectively. Thus, we believe that each participant had an equal probability of being selected at the urban and rural levels because population and prevalence had similar effects on sample size from these selected groups.

The sample size required for this study was calculated based upon the prevalence of DM in the absence of details on the health literacy variable. The prevalence of DM in urban or rural residents in Gansu Province was 11.5 or 9.5%, respectively (8).


$$\begin{array}{ll} n & =n_{c}/(1+n_{c}/N);\\ n_{c} & =Z_{\alpha /2}{}^{2}\times p\times (1-p)/d^{2} \end{array}$$

where *n* = sample size, *n*_*c*_ = sample size before finite population correction, *Z*_α/2_ = 1.96 (confidence level), *p* = prevalence of diabetes, *d* = 5% (tolerated margin of error), and *N* = 320,000 (estimated average population of a district/county in Gansu Province).

The calculation yields a survey sample size of at least 193 in each district and 163 in each county. Thus, the total sample size was *n* = 1,424.

### Questionnaire Development and Content

The questionnaire was designed according to the Guidelines for the Prevention and Treatment for Diabetes, China (2017) (23). The scales that are referenced are derived from published papers in China and are widely used as follows: knowledge, attitude, practice assessment scale for diabetes patients (26), and the community health literacy scale for diabetes (27). The contents included are as follows: (1) personal information; (2) disease information; and (3) diabetes health literacy level. In more detail, there were 37 items within six aspects with a total score of 47 points, relating to diabetes health knowledge, attitude and practice, including practice habits, dietary therapy, exercise therapy, drug therapy, blood glucose and blood pressure monitoring, and hypoglycaemia prevention. In relation to specific usage of the internet to access health information, there were two closely related questions in our questionnaire, namely, patients with DM were first asked if they had internet access; if the answer was yes, then the immediately following, second, the question was—how often and for how long has internet surfing been utilized for accessing health information about diabetes?

Each question in the diabetes health knowledge scale was assigned 1 point for each correct answer and 0 for each incorrect or unclear answer (28). The health attitude scale adopted Likert's type 5-level equidistance bidirectional semantic quantifiers (28), i.e., very important, important, general, not important, or not very important. To maintain the unity of the maximum score assigned to the knowledge scale, the values were successively assigned as 3, 2.4, 1.8, 1.2, and 0.6, respectively. The health practice scale utilizes 2-levels of response options (“Yes”, “No”) and 5-level response options (“Never”, “Occasionally”, “Sometimes”, “Often”, “Always”), respectively. The former was assigned a value of 1 for “Yes” and 0 for “No”, whereas the latter was assigned values of 1, 0.8, 0.6, 0.4, and 0.2, respectively (28). When the scaled score was less than or equal to the average score, it is considered to be a poor score (29).

The Cronbach's α values were 0.83, 0.80, 0.94, or 0.78 for the total health literacy scale, knowledge, attitude, or practice scale, respectively.

### Statistical Analysis

Double input was used in Epidata 3.1 to establish a database. All the data were analyzed by SPSS software. All continuous variables were denoted as mean ± SD and median (inter-quartile range), whereas categorical variables were described as counts (*n*) and percentage (%). The factors that influenced the health literacy level and internet accessibility were analyzed by logistic regression bivariate and multivariate analysis, whereas the chi-square test was used to compare the differences in the needs of patients with DM. All the statistical analyses were two-sided, and *P* ≤ 0.05 was considered statistically significant.

## Results

### Factors Influencing Health Literacy Concerning Diabetic Information for Patients With DM

**Figure 2:**
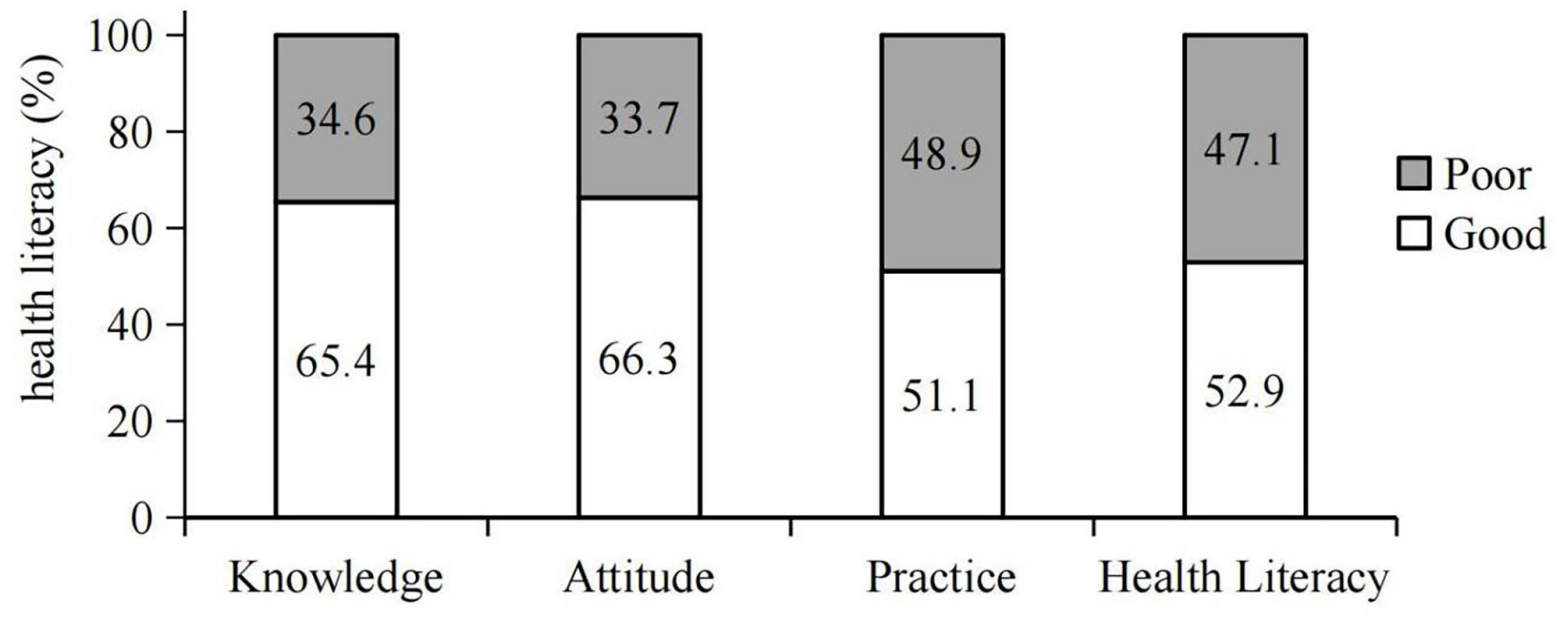
Level of health literacy regarding diabetes among the respondents.

A total of 1,563/1,633 (95.7%) valid questionnaires were collected in the current study. Among these, 875 (56.0%) were female, the mean age was 65.9 ± 9.7 years, 1,446 (92.5%) were Han race, 910 (58.2%) lived in urban areas, 1,364 (87.3%) were married, 1,460 (93.4%) lived with their families, 790 (50.5%) were farmers and workers, 661 (42.3%) had suffered from DM for a period ≥ 5 years, 322 (20.6%) had a family history of DM, and 840 (53.7%) had internet access (Table 1). Our data demonstrate that the knowledge, attitude, practice, and health literacy about diabetes in patients with DM were 65.4, 66.3, 51.1, or 52.9%, respectively (Figure 2).

**Table 1:** Bivariate and multivariate analyses of factors associated with health literacy level among the respondents [*n*, (%)].

**Variables**	**Categories**	**Health literacy**		***COR*** **(95% *CI*)**	***P***	***AOR*** **(95% *CI*)**	***P***
		**Good**	**Poor**				
Sex	Male	399 (58.0)	289 (42.0)	0.69 (0.57, 0.85)	<0.001	1.21 (0.95, 1.56)	NS
	Female	428 (48.9)	447 (51.1)	1.00		1.00	
Age (Yr)	<55	100 (56.8)	76 (43.2)	0.68 (0.47, 0.99)	0.046	0.70 (0.45, 1.09)	NS
	55~	246 (57.3)	183 (42.7)	0.67 (0.50, 0.90)	0.008	0.82 (0.57, 1.16)	NS
	65~	340 (51.5)	320 (48.5)	0.85 (0.64, 1.11)	NS	0.79 (0.58, 1.08)	NS
	75~	141 (47.3)	157 (52.7)	1.00		1.00	
Race	Han	765 (52.9)	681 (47.1)	0.68 (0.41, 1.11)	NS	0.92 (0.54, 1.61)	NS
	Hui	33 (66.0)	17 (34.0)	0.39 (0.18, 0.84)	0.016	0.64 (0.28, 1.50)	NS
	Other ethnic	29 (43.3)	38 (56.7)	1.00		1.00	
Resident	Urban	553 (60.8)	357 (39.2)	0.47 (0.38, 0.57)	<0.001	0.85 (0.62, 1.16)	NS
	Rural	274 (42.0)	379 (58.0)	1.00		1.00	NS
Marital status	Married	750 (55.0)	614 (45.0)	0.49 (0.35, 0.68)	<0.001	0.78 (0.51, 1.18)	NS
	Unmarried/divorced/separated	13 (46.4)	15 (53.6)	0.69 (0.31, 1.54)	NS	1.36 (0.56, 3.31)	NS
	Widowed	64 (37.4)	107 (62.6)	1.00		1.00	NS
Living status	Live alone	41 (39.8)	62 (60.2)	1.76 (1.17, 2.65)	0.006	1.34 (0.80, 2.23)	NS
	Live with family members	786 (53.8)	674 (46.2)	1.00			NS
Working status	Farmers/workers	333 (42.2)	457 (57.8)	1.56 (1.11, 2.19)	0.011	0.77 (0.50, 1.17)	NS
	Enterprise/business personnel	73 (76.8)	22 (23.2)	0.34 (0.19, 0.60)	<0.001	0.47 (0.25, 0.87)	0.017
	Merchant/service personnel	18 (64.3)	10 (35.7)	0.63 (0.27, 1.45)	NS	1.01 (0.41, 2.44)	NS
	Retired	318 (64.9)	172 (35.1)	0.61 (0.43, 0.88)	0.008	0.70 (0.46, 1.07)	NS
	Others	85 (53.1)	75 (46.9)	1.00		1.00	
Education	≤Primary school	308 (39.3)	475 (60.7)	4.10 (2.61, 6.43)	<0.001	1.59 (0.92, 2.75)	NS
	Primary and middle school	247 (65.3)	131 (34.7)	1.41 (0.87, 2.27)	NS	1.01 (0.59, 1.71)	NS
	High school/technical school	195 (65.9)	101 (34.1)	1.38 (0.84, 2.25)	NS	1.09 (0.64, 1.85)	NS
	≥University	77 (72.6)	29 (27.4)	1.00		1.00	
Monthly income (¥)	<500	198 (32.9)	404 (67.1)	4.57 (3.48, 6.02)	<0.001	2.06 (1.35, 3.14)	0.001
	500~	104 (60.1)	69 (39.9)	1.49 (1.03, 2.16)	0.037	0.99 (0.63, 1.57)	NS
	2,000~	256 (64.2)	143 (35.8)	1.25 (0.93, 1.69)	NS	1.05 (0.75, 1.47)	NS
	3,500~	269 (69.2)	120 (30.8)	1.00		1.00	
Course of DM (Yr)	<5	308 (46.6)	353 (53.4)	1.64 (1.30, 2.07)	<0.001	1.68 (1.29, 2.20)	<0.001
	5~	220 (55.8)	174 (44.2)	1.31 (0.87, 1.48)	NS	1.26 (0.93, 1.70)	NS
	10~	299 (58.9)	209 (41.1)	1.00		1.00	
BMI (kg/m^2^)	<24	355 (50.9)	343 (49.1)	1.06 (0.78, 1.44)	NS	1.24 (0.89, 1.74)	NS
	≥24	359 (55.3)	290 (44.7)	0.89 (0.65, 1.21)	NS	1.09 (0.78, 1.53)	NS
	≥28	113 (52.3)	104 (47.7)	1.00		1.00	NS
Family history of DM	Yes	186 (57.8)	136 (42.2)	0.86 (0.56, 1.34)	NS	0.88 (0.54, 1.42)	NS
	No	582 (51.4)	550 (48.6)	1.12 (0.75, 1.65)	NS	0.87 (0.56, 1.34)	NS
	Unknown	59 (51.0)	50 (49.0)	1.00		1.00	
Access internet	Yes	572 (68.1)	268 (31.9)	0.26 (0.21, 0.32)	<0.001	0.35 (0.28, 0.44)	<0.001
	No	255 (35.3)	468 (64.7)	1.00		1.00	

There were significant correlations between health literacy and different sex, age, residence, marital status, living status, working status, educational level, monthly income, course of DM, and internet access by bivariate analysis. Using multivariate analysis, enterprise or business personnel (*P* < 0.05), high income (>¥2000), disease duration (>5 years), and internet access were protective factors for health literacy (Table 1), all of them, *P* < 0.001.

### Factors Influencing Internet Access to Obtain Diabetic Information for Patients With DM

**Figure 3:**
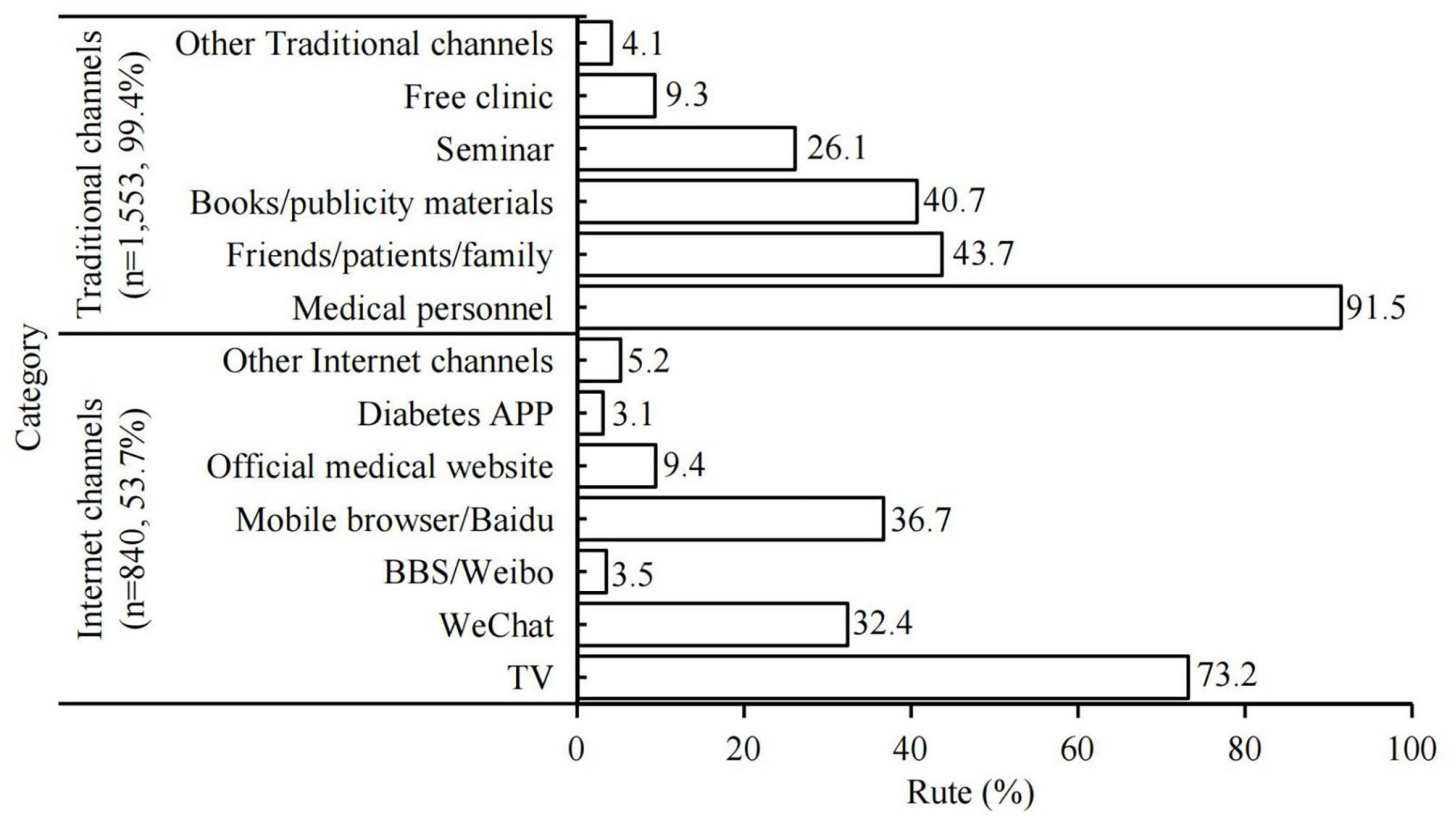
Current status of access to health information for the respondents.

A total of 1,553 (99.4%) of patients with DM were able to obtain diabetic information from traditional sources, mainly through medical personnel (*n* = 1,421, 91.5%), followed by friends, other patients, and family (*n* = 679, 43.7%) and books (*n* = 632, 40.7%). On the other hand, there were 840 (53.7%) of patients with DM who obtained health information from the internet, e.g., TV (*n* = 615, 73.2%), mobile phone/computer browsing (*n* = 308, 36.7%), or WeChat (*n* = 272, 32.4%) (Figure 3).

There was a significant correlation between internet access and sex, age, residence, marital status, living status, working status, education or monthly income, and family history of DM in patients with DM, using bivariate analysis. On multivariate analysis, age < 60 years (*P* < 0.05) was a protective factor for internet access, and living in rural areas (*P* < 0.001); farmers or workers (*P* < 0.01); retirement (*P* < 0.001), education level below primary school (*P* < 0.001), middle school, or secondary school (*P* < 0.05); and income <¥2,000 (*P* < 0.05) were risk factors for internet access (Table 2).

**Table 2:** Bivariate and multivariate analyses of factors associated with the internet accessibility among the respondents [*n*, (%)].

**Variables**	**Categories**	**Access internet**		***COR*** **(95% *CI*)**	***P***	***AOR*** **(95% *CI*)**	***P***
		**Yes**	**No**				
Sex	Male	420 (61.0)	268 (39.0)	0.59 (0.48, 0.72)	<0.001	1.13 (0.88, 1.45)	NS
	Female	420 (48.0)	455 (52.0)	1.00		1.00	
Age (Yr)	<55	116 (65.9)	60 (34.1)	0.42 (0.29, 0.62)	<0.001	0.52 (0.33, 0.82)	0.005
	55~	271 (63.2)	158 (36.8)	0.48 (0.35, 0.64)	<0.001	0.56(0.39, 0.79)	0.001
	65~	319 (48.3)	341 (51.7)	0.87 (0.66, 1.15)	NS	0.74 (0.54, 1.01)	NS
	75~	134 (45.0)	164 (55.0)	1.00		1.00	
Race	Han	771 (53.3)	675 (46.7)	1.15 (0.70, 1.88)	NS	1.58 (0.89, 2.82)	NS
	Hui	31 (62.0)	19 (38.0)	0.80 (0.38, 1.70)	NS	1.41 (0.60, 3.30)	NS
	Other ethnic	38 (56.7)	29 (43.3)	1.00		1.00	
Resident	Urban	533 (58.6)	377 (41.4)	0.63 (0.51, 0.77)	<0.001	1.89 (1.34, 2.64)	<0.001
	Rural	307 (47.0)	346 (53.0)	1.00		1.00	
Marital status	Married	760 (55.7)	604 (44.3)	0.42 (0.30, 0.58)	<0.001	0.80 (0.52, 1.22)	NS
	Unmarried/divorced/separated	21 (75.0)	7 (25.0)	0.18 (0.07, 0.44)	<0.001	0.39 (0.14, 1.06)	NS
Living type	Widowed	59 (34.5)	112 (65.5)	1.00		1.00	
	Live alone	41 (39.8)	62 (60.2)	1.83 (1.22, 2.75)	0.004	1.38 (0.81, 2.35)	NS
	Live with family members	799 (54.7)	661 (45.3)	1.00		1.00	
Working status	Farmers/workers	342 (43.3)	448 (56.7)	2.57 (1.80, 3.67)	<0.001	2.08 (1.32, 3.28)	0.002
	Enterprise/business personnel	77 (81.1)	18 (18.9)	0.46 (0.25, 0.84)	0.012	1.22 (0.61, 2.43)	NS
	Merchant/service personnel	22 (78.6)	6 (21.4)	0.54 (0.21, 1.40)	NS	1.81 (0.65, 5.06)	NS
	Retired	293 (59.8)	197 (40.2)	1.32 (0.91, 1.92)	NS	2.83 (1.78, 4.51)	<0.001
	Others	106 (66.3)	54 (33.8)	1.00		1.00	
Education	≤Primary school	305 (39.0)	478 (61.0)	5.98 (3.66, 9.78)	<0.001	3.36 (1.88, 6.01)	<0.001
	Middle school	252 (66.7)	126 (33.3)	1.91 (1.14, 3.20)	0.014	1.67 (0.95, 2.94)	NS
	High school/technical school	199 (67.2)	97 (32.8)	1.86 (1.10, 3.16)	0.021	1.82 (1.03, 3.20)	0.039
	≥University	84 (79.2)	22 (20.8)	1.00		1.00	
Monthly income (¥)	<500	186 (30.9)	416 (69.1)	5.75 (4.34, 7.61)	<0.001	6.34 (4.10, 9.81)	<0.001
	500~	112 (64.7)	61 (35.3)	1.40 (0.95, 2.05)	NS	1.80 (1.11, 2.91)	0.017
	2,000~	262 (65.7)	137 (34.3)	1.34 (0.99, 1.82)	NS	1.16 (0.82, 1.63)	NS
	3,500~	280 (72.0)	109 (28.0)	1.00		1.00	
Course of DM (Yr)	<5	346 (52.3)	315 (47.7)	0.99 (0.78, 1.24)	NS	0.91 (0.69, 1.19)	NS
	5~	230 (58.4)	164 (41.6)	0.77 (0.59, 1.01)	NS	0.79 (0.59, 1.08)	NS
	10~	264 (52.0)	244 (48.0)	1.00		1.00	
BMI (Kg/m^2^)	<24	373 (53.4)	325 (46.6)	0.82 (0.61, 1.12)	NS	0.92 (0.65, 1.29)	NS
	≥24	362 (55.8)	287 (44.2)	0.75 (0.55, 1.02)	NS	0.92 (0.65, 0.31)	NS
	≥28	105 (48.6)	111 (51.4)	1.00		1.00	
Family history of DM	Yes	194 (60.2)	128 (39.8)	1.52 (0.95, 2.42)	NS	1.71 (1.01, 2.87)	0.044
	No	570 (50.4)	562 (49.6)	2.27 (1.49, 3.47)	<0.001	2.23 (1.39, 3.58)	0.001
	Unknown	76 (71.0)	33 (29.0)	1.00		1.00	

### The Expectations Concerning Internet Diabetic Information Among Patients With DM

Among the respondents, 1,465 (93.7%) expected to obtain relevant information from traditional sources, e.g., doctors. Similarly, information sourced from other medical personnel also had a relatively high expectation (*n* = 397, 27.1%), followed by books, publicity materials or published periodicals (*n* = 143, 9.8%), or seminars (*n* = 110, 7.5%). Compared with those patients without access to the internet, patients with DM with internet access had a lower expectation to obtain relevant DM-related information from traditional sources (*P* < 0.05) (Table 3).

**Table 3:** Respondents' expectations of web-related information [*n*, (%)].

**Variables**		**Access internet**		***χ^2^***	***P***
		**Yes**	**No**		
Traditional sources (*n* = 1,465)	Doctors and other medical personnel	122 (30.7)	275 (69.3)	113.36	<0.001
	Friends/colleagues/patients	45 (50.0)	45 (50.0)	0.54	NS
	Family members	30 (39.0)	47 (61.0)	7.12	0.008
	Books/publicity materials/publicity column	69 (48.3)	74 (51.7)	1.91	NS
	Seminar	55 (50.0)	55 (50.0)	0.67	NS
Internet sources (*n* = 1,145)	TV	560 (63.3)	324 (36.7)	75.53	<0.001
	Mobile website	268 (90.2)	29 (9.8)	196.43	<0.001
	WeChat	234 (85.7)	39 (14.3)	136.01	<0.001
	Official medical website	184 (82.1)	40 (17.9)	84.83	<0.001
	Diabetes APP	66 (81.5)	15 (18.5)	26.44	<0.001
	BBS/Microblogy	34 (87.2)	5 (12.8)	17.99	<0.001
Resource type (*n* = 1,193)	Video clip	580 (63.2)	338 (36.8)	79.71	<0.001
	Webpage	84 (77.8)	24 (22.2)	26.96	<0.001
	Picture	178 (67.7)	85 (32.3)	24.71	<0.001
	Experts' information/text	424 (79.5)	109 (20.5)	216.68	<0.001
	BBS/Microblog	45 (81.8)	10 (18.2)	18.08	<0.001
Content of requirements (*n* = 1,385)	Causes of disease	505 (66.4)	255 (33.6)	96.05	<0.001
	Psychological education	388 (80.8)	92 (19.2)	204.51	<0.001
	Dietary guidelines	632 (56.4)	489 (43.6)	11.08	0.001
	Exercise guidance	477 (52.2)	436 (47.8)	1.98	NS
	Drug treatment	448 (52.6)	403 (47.4)	0.91	NS
	Monitoring	240 (67.2)	117 (32.8)	33.839	<0.001
	Prevention and treatment of complications	371 (59.1)	257 (40.9)	12.01	0.001

In contrast, there were 1,145 (73.3%) respondents who expected to obtain relevant information through internet sources. A TV broadcast *via* broadband was the first choice (*n* = 884, 77.2%). While among the resource forms of information output, 918 (76.9%) expected to view video clips, followed by information and text of the experts (*n* = 533, 44.7%). There was a positive correlation between internet accessibility and the demand for obtaining relevant information (*P* < 0.05) (Table 3).

There were 1,385 (88.6%) respondents who expected to acquire diabetes-related knowledge, among which dietary guidelines was the highest expectation (*n* = 1,121, 80.9%), followed by the need for blood-glucose monitoring (*n* = 357, 25.8%) and psychological education (*n* = 480, 34.7%). The comparison between the groups with and without internet accessibility revealed that there was no significant difference in terms of exercise guidance and drug treatment (Table 3).

### Compliance With Monitoring Behavior Amongst Patients With DM

The recommendation for monitoring blood glucose is four times/month for most of the patients with DM in China, according to The Chinese Guidelines for the Prevention and Treatment of Diabetes (21). In the current study, however, only 34.9% of patients with DM complied with the guidelines. In terms of monitoring behavior, males, urban patients, merchant/service personnel, education till middle school and above, monthly income >¥3,500, disease course, and family history of DM were positively related to monitoring behavior for patients with diabetes (*P* < 0.05) (Table 4).

**Table 4:** Monitoring behavior and internet trust status of the respondents [*n*, (%)].

**Variables**	**Categories**	**Self-monitoring**			**Trust of the internet**		
		**≥4 times/month**	**<4 times/month**	***χ^2^*/*P***	**Full trust**	**Partial trust and distrust**	***χ^2^*/*P***
Sex	Male	263 (38.2)	425 (61.8)	6.10/0.014	117 (27.9)	303 (72.1)	0.02/0.877
	Female	282 (32.2)	593 (67.8)		115 (27.4)	305 (72.6)	
Age (years)	<55	79 (44.9)	97 (55.1)	15.07/<0.001^a^	29 (25.0)	87 (75.0)	0.06/0.806^a^
	55~	168 (39.2)	261 (60.8)		80 (29.5)	191 (70.5)	
	65~	207 (31.4)	453 (68.6)		84 (26.3)	235 (73.7)	
	75~	91 (30.5)	207 (69.5)		39 (29.1)	95 (70.9)	
Race	Han	495 (34.2)	951 (65.8)	4.51/0.105	216 (28.0)	555 (72.0)	2.14/0.343
	Hui	24 (48.0)	26 (52.0)		5 (16.1)	26 (83.9)	
	Other ethnic	26 (38.8)	41 (61.2)		11 (28.9)	27 (71.1)	
Resident	Urban	357 (39.2)	553 (60.8)	18.25/<0.001	131 (24.6)	402 (75.4)	6.75/0.009
	Rural	188 (28.8)	465 (71.2)		101 (32.9)	206 (67.1)	
Marital status	Unmarried	4 (40.0)	6 (60.0)	5.30/0.151	7 (70.0)	3 (30.0)	9.88/0.020
	Married	486 (35.6)	878 (64.4)		205 (27.0)	555 (73.0)	
	Divorced/separated	8 (44.4)	10 (55.6)		2 (18.2)	9 (81.8)	
	Widowed	47 (27.5)	124 (72.5)		18 (30.5)	41 (69.5)	
Living type	Live alone	35 (34.0)	68 (66.0)	0.04/0.845	5 (12.2)	36 (87.8)	2.13/0.024
	Live with family members	510 (34.9)	950 (65.1)		227 (28.4)	572 (71.6)	
Working status	Farmers/workers	242 (30.6)	548 (69.4)	13.24/0.010	112 (32.7)	230 (67.3)	12.04/0.017
	Enterprise/business personnel	36 (37.9)	59 (62.1)		24 (31.2)	53 (68.8)	
	Merchant/service personnel	12 (42.9)	16 (57.1)		7 (31.8)	15 (68.2)	
	Retired	189 (38.6)	301 (61.4)		61 (20.8)	232 (79.2)	
	Others	66 (41.3)	94 (58.8)		28 (26.4)	78 (73.6)	
Education	Primary school and below	209 (26.7)	574 (73.3)	32.03/<0.001^a^	92 (30.2)	213 (69.8)	0.40/0.530^a^
	Middle school	164 (43.4)	214 (56.6)		62 (24.6)	190 (75.4)	
	High school/technical school	128 (43.2)	168 (56.8)		55 (27.6)	144 (72.4)	
	University and above	44 (41.5)	62 (58.5)		23 (27.4)	61 (72.6)	
Monthly income (¥)	<500	160 (26.6)	442 (73.4)	36.51/<0.001^a^	66 (35.5)	120 (64.5)	8.77/0.003^a^
	500~	57 (32.9)	116 (67.1)		42 (37.5)	70 (62.5)	
	2,000~	156 (39.1)	243 (60.9)		50 (19.1)	212 (80.9)	
	3,500~	172 (44.2)	217 (55.8)		74 (26.4)	206 (73.6)	
course of DM (Yr)	<5	187 (28.3)	474 (71.7)	18.87/<0.001^a^	99 (28.6)	247 (71.4)	2.95/0.229
	5~	154 (39.1)	240 (60.9)		70 (30.4)	160 (69.6)	
	10~	204 (40.2)	304 (59.8)		63 (23.9)	201 (76.1)	
BMI (Kg/m^2^)	<24	238 (34.1)	460 (65.9)	0.58/0.445^a^	92 (24.7)	281 (75.3)	4.80/0.091^a^
	≥24	227 (35.0)	422 (65.0)		103 (28.5)	259 (71.5)	
	≥28	80 (37.0)	136 (63.0)		37 (35.2)	68 (64.8)	
Family history of DM	Yes	127 (39.4)	195 (60.6)	8.42/0.015	52 (26.8)	142 (73.2)	2.93/0.231
	No	371 (32.8)	761 (67.2)		165 (28.9)	405 (71.1)	
	Unclear	47 (53.1)	62 (56.9)		15 (19.7)	61 (80.3)	

a *Trend chi-square test statistics*.

### Trustworthiness of Internet Health Information Amongst Patients With DM

In terms of the blood glucose monitoring level, males, urban patients, merchant/service personnel, education until middle school or above, monthly income (¥) >3,500, disease course, and family history of DM were positively related to the frequency of monitoring for patients with diabetes (*P* < 0.05) (Table 4).

Thus, monthly income, occupation, district of residence, marital status, and residential type were also found to be factors influencing trust of internet-derived information. However, living in an urban area and high monthly income were found to be inversely related to the trust of the internet-sourced information (Table 4).

## Discussion

In the current study, we found that the health literacy of patients with DM in Gansu remained at a reasonable level compared with others in China (30), and these patients had a positive attitude toward the prevention and treatment of diabetes. Interestingly, we also observed that neither the understanding of relevant prevention and treatment knowledge was sufficiently comprehensive for the patients with DM, nor was the knowledge in self-management. However, the health literacy of patients with DM who engaged in proactive internet surfing was higher than that of those without internet access, and more patients with DM agreed that they would like to obtain health information from an internet source than those who have already obtained information through the internet.

Our data demonstrate that slightly >50% of patients with DM possess adequate DM health literacy in Gansu Province, China, which is substantially lower compared with those patients with DM in the developed countries (90%) (31). The big difference between our current finding (50%) and others within the developed countries (90%) may be due to the timing of the establishment/development of internet infrastructure, which has been much early in the developed countries; whereas the development of the internet has occurred at least a decade later in Gansu, China (32). Moreover, Gansu Province has the lowest economic GDP in China, which would substantially impact business and education, and also internet infrastructure, compared with the highest GDP regions in China (e.g., Shanghai or Beijing) (33). This may explain the lower DM health literacy in Gansu Province, most likely due to economic status.

There was no significant correlation between health literacy and sex, occupation, or residence in Gansu Province, although a correlation was found for education, which contrasts with others (29). Such differences may be related to different sample sizes, subject sources, and questionnaires used between our study and others. People with higher levels of education are easily able to adapt to new knowledge, which also could be utilized by the patients with DM (29), whereas the patients with DM in Gansu had a relatively low standard of education and poor health literacy, which is consistent with Gansu Province having the lowest GDP in all of China.

In addition, our data demonstrate that improved DM literacy in those participants with DM for >5 years was most likely due to these patients with DM who were keen to obtain DM-related information as extensively as possible from any resource (34). This is supported by others, showing that the longer the disease course in patients with DM, the more active patients seek the help of clinicians and improve their health literacy level (35). Apart from the different regions/nations in which the comparable research was conducted, our current study showed that patients with DM had variable access to DM information, as well as health education, based on their residency in urban or rural communities. There is a correlation between health literacy level and management in DM (36). Thus, it is desirable to encourage and promote patients with DM, especially in rural areas, to improve their health literacy with the objective being to either prevent or minimize the progression of DM.

Furthermore, we found that there is a close correlation between access to the internet and socioeconomic status within Gansu Province, i.e., internet access may be considered as a proxy indictor for socioeconomic status. We have observed that some variables related to socioeconomic status were significantly different between patients with DM with and without access to the internet in the current study. This is supported by our finding that some patients with DM from rural regions, especially among the relatively old participants, have had limited and/or no access to the internet, either due to unwillingness to acquire new technology and/or cost constraints associated with low income preventing access to the internet. Thus, this cohort could only obtain DM-related information from traditional routes and consequently had a relatively poor health literacy. It has been reported that health information obtained through internet access helps patients with DM to improve their self-management utilizing objective judgment concerning their health condition (37).

However, internet access has not been affordable and accessible in these rural regions over the last few decades, particularly in the relatively mountainous regions in Gansu. Consequently, there has been a substantial delay for the infrastructure development in these regions, mainly because the GDP of Gansu Province is the lowest in China. However, the contribution from the local authority to improve internet accessibility, including improving the infrastructure within the internet pathway *via* ground and/or satellite, has been increased, particularly within the last 10 years. In addition, the substantial cost for the internet is being subsidized by the local authorities (38), which has enabled at least some of these patients, if not all, to utilize internet surfing. Additional direct input from the local authorities includes the implementation of mandatory education till 9 years, which has substantially improved general literacy, *via* subsidizing educational costs, thereby avoiding students suspending their education while they are still only semiliterate for financial and/or social reasons. A specific initiative instituted through the most popular and ubiquitous Chinese form of social media, WeChat, has been active in the distribution of a large number of video clips providing detailed explanations pertaining to a range of DM issues from semiofficial channels. Furthermore, the local health authority periodically reminds the community health centers of the need to continue to inform their patients with DM to monitor blood glucose and to disseminate advice on diet and exercise, as well as the general management of DM, utilizing local WeChat groups established and managed specifically for patients with DM.

Additionally, our data demonstrate that positive attitude or practice amongst patients with DM was only 66.3 or 51.1%, respectively, suggesting that attitudes might not always translate to practice (39). Previous trials of health literacy and DM demonstrate that there is a higher efficacy associated with internet-provided health education compared with that from traditional routes (40). Our current study provided a thorough approach with more insights to confirm the previous preliminary data by providing more detailed solid evidence to support these findings. More specifically, our study demonstrated that health education was improved *via* the internet, particularly in rural areas of Gansu Province, China.

The current study has been able to determine/compare how widely internet accessibility and affordability affect health literacy outcomes among the different regions with different socioeconomic statuses. Our findings are consistent with others, showing that improved accessibility to the internet is able to provide better health education (40), particularly during the current COVID-19 environment with significant curtailment of face-to-face education opportunities (41).

Trust is fundamentally important in the management of DM from both the physical and psychological points of view (42). We found that only 27.6% of patients with DM with access to the internet fully trusted online information. The main concerns are related to conflicts of interest or bias from commercial sources (43). Thus, the resources located within the media should be managed and endorsed more tightly by the local health authority to ensure the provision of high-quality health information on various media platforms and to standardize the resources available online, particularly for DM health information.

Moreover, we found that patients with DM had a high desire for diet and exercise management, but low motivation objectively for monitoring behavior, i.e., only 34.9% of the respondents complied with the standard monitoring frequency (four times/month) (23), which may explain the subsequent consequence of poor management of DM.

Finally, we found that the patients with DM who engaged in good monitoring were correlated with sex, age, residential type, occupation, education, monthly income, disease course, and family history of DM. This finding further supports the conclusion that these patients with high socioeconomic status are likely to be more willing to take care of their health status more frequently. Our data are consistent with others (44), showing that less than one-third of the patients achieved the minimum blood glucose monitoring criteria.

Consideration needs to be given to the potential of the pandemic of COVID-19 to interfere with this study. The current study was performed in July 2020 in China, when the pandemic of COVID-19 was under reasonably good control in China, particularly in the rural regions. Thus, the lockdown had been lifted several months prior to the initiation of our investigation and had no/or limited impact on the current study. Although there had been no COVID-19 cases for more than 4 months in Gansu Province prior to the commencement of the study, participants within the study still abided by the relevant regulations on epidemic prevention and control during the investigation, including measuring body temperature, wearing facial masks by investigators and respondents, and maintaining social distancing between all participants.

There are limitations in the current study, for example, it would be more appropriate to compare a group of patients with DM who enjoyed better health literacy *via* internet access vs. a similar group of patients with DM who had no access to the internet. However, this study design is beyond the scope of the research team at the current stage but will be investigated in the future.

In addition, the current study was a self-rated assessment, which will inevitably include some subjective factor(s). A direct comparison between cohorts with similar socioeconomic status is not applicable; however, a future study will be specifically designed to explore this issue using an RCT/quasi-experimental approach, which would allow for a study in which there is a more controllable approach for demonstrating the causative factors among the participants.

Finally, there is a potential bias in the current study, e.g., a recall and/or desirability bias. Though the investigators were trained uniformly before the study, there were differences in their capacity to obtain accurate information, such as especially for those patients with DM for a long period of time, which are more likely to have had such bias.

## Conclusion

We conclude that socioeconomic status and access to the internet were the main contributing factors for health literacy, as socioeconomic status is closely related to access to the internet. Furthermore, there is a positive correlation between internet accessibility and health literacy in patients with DM.

## Data Availability Statement

The original contributions presented in the study are included in the article/supplementary material, further inquiries can be directed to the corresponding authors.

## Ethics Statement

The studies involving human participants were reviewed and approved by Ethical Review Committee of Affiliated Hospital of Gansu University of Chinese Medicine. The patients/participants provided their written informed consent to participate in this study.

## Author Contributions

NZ and JF conceived the proposed idea. XL, WP, and XZ participated in the methodology and research mentioned in this study. NZ, HZ, and RD collected and synthesized the data. WP and XZ encouraged and supervised the results of this study. NZ and XL analyzed the investigation data and obtained the results. BH, SB, and JF revised the manuscript. All authors discussed the results, contributed to the final manuscript, and agreed on the final version.

## Funding

This study was supported by the 2019 Grant-in-Aid for Grassroots Basic Public Health Services from the Health and Wellness Commission of Gansu Province (Grant Number 31140215), Gansu Provincial Administration of Traditional Chinese Medicine Major Science and Technology Special Project (Grant Number GZKZD-2018-01), 2020 Science and Technology Project of Chengguan District, Lanzhou (Grant Number 2020-2-11-16), and the Graduate Innovation Fund Project of Gansu University of Chinese Medicine (Grant Number CX2020-59).

## Conflict of Interest

The authors declare that the research was conducted in the absence of any commercial or financial relationships that could be construed as a potential conflict of interest.

## Publisher's Note

All claims expressed in this article are solely those of the authors and do not necessarily represent those of their affiliated organizations, or those of the publisher, the editors and the reviewers. Any product that may be evaluated in this article, or claim that may be made by its manufacturer, is not guaranteed or endorsed by the publisher.
